# Sex differences in immune gene expression in the brain of a small shorebird

**DOI:** 10.1007/s00251-022-01253-w

**Published:** 2022-01-27

**Authors:** José O. Valdebenito, Kathryn H. Maher, Gergely Zachár, Qin Huang, Zhengwang Zhang, Larry J. Young, Tamás Székely, Pinjia Que, Yang Liu, Araxi O. Urrutia

**Affiliations:** 1 Milner Centre for Evolution, Department of Biology and Biochemistry, University of Bath, Bath, UK; 2 Department of Evolutionary Zoology and Human Biology, University of Debrecen, Debrecen, Hungary; 3 Department of Animal and Plant Sciences, University of Sheffield, Sheffield, UK; 4 Ministry of Education Key Laboratory for Biodiversity Sciences and Ecological Engineering, College of Life Sciences, Beijing Normal University, Beijing, China; 5 Department of Anatomy, Histology and Embryology, Semmelweis University, Budapest, Hungary; 6 State Key Laboratory of Biocontrol, School of Ecology, Sun Yat-Sen University, Guangzhou, China; 7 Silvio O. Conte Center for Oxytocin and Social Cognition, Center for Translational Social Neuroscience, Department of Psychiatry and Behavioral Sciences, Yerkes National Primate Research Center, Emory University, Atlanta, USA; 8 Instituto de Ecología, UNAM, Ciudad de México, Mexico

**Keywords:** Sex-biased gene expression, Avian immunity, RNA-seq, Brain, Mating system

## Abstract

Males and females often exhibit differences in behaviour, life histories, and ecology, many of which are typically reflected in their brains. Neuronal protection and maintenance include complex processes led by the microglia, which also interacts with metabolites such as hormones or immune components. Despite increasing interest in sex-specific brain function in laboratory animals, the significance of sex-specific immune activation in the brain of wild animals along with the variables that could affect it is widely lacking. Here, we use the Kentish plover (*Charadrius alexandrinus*) to study sex differences in expression of immune genes in the brain of adult males and females, in two wild populations breeding in contrasting habitats: a coastal sea-level population and a high-altitude inland population in China. Our analysis yielded 379 genes associated with immune function. We show a significant male-biased immune gene upregulation. Immune gene expression in the brain did not differ in upregulation between the coastal and inland populations. We discuss the role of dosage compensation in our findings and their evolutionary significance mediated by sex-specific survival and neuronal deterioration. Similar expression profiles in the coastal and inland populations suggest comparable genetic control by the microglia and possible similarities in pathogen pressures between habitats. We call for further studies on gene expression of males and females in wild population to understand the implications of immune function for life-histories and demography in natural systems.

**Supplementary information:**

The online version contains supplementary material available at 10.1007/s00251-022-01253-w.

## Introduction

Sex differences in behaviour, morphology, and physiology are widespread in nature, with most dioecious animals thought to present at least some degree of sexual dimorphism. The brain — the centre of the nervous system in many organisms — is an example of one of these sexually dimorphic structures as it may differ in size, function and gene expression (Biswal et al. 2010; DeVoogd and Székely 1998; Kang et al. 2011). Sex differences (or sexual dimorphism as is usually termed) in the brain are being increasingly investigated using humans and laboratory animals. Although in wild species these topics have been considerably less explored, recent evidence suggests sex differences in neural organisation and gene expression in the brain (Catalán et al. 2012; Delage and Cornil 2020; Naurin et al. 2011; Rotllant et al. 2017; Sharma et al. 2014; Yang et al. 2006).

With evidence suggesting the importance of functional and structural sex differences in the brain, it is possible that complementary processes such as disease risk and its defence response may also differ between the sexes. Indeed, sex differences are found in neuronal diseases such as Parkinson’s disease, where males exhibit a greater reduction in global cognition and language than females, or in Alzheimer disease, presenting women with faster rates of brain atrophy than males (Bakeberg et al. 2021; Ferretti et al. 2018). Understanding the causes of these differences are important, since many diseases — including the recent COVID-19 — have different mortality rates in males and females, ultimately resulting in sex-biased mortalities (e.g. Lemaître et al. 2020; Pipoly et al. 2015). Sex differences in microglial and astrocytic cells, such as their heightened sensitivity to inflammatory stimuli and their anatomical distribution (Lenz et al. 2013; Martin-Jiménez et al. 2019; Schwarz et al. 2012; Siani et al. 2017; Villa et al. 2018), have been postulated to mediate sex differences in cognition and memory in rodent models (Chamniansawat and Sawatdiyaphanon 2018; Yun et al. 2018). However, research addressing the main system responsible for inflammatory responses and pathogen defence, i.e., the immune system, in the brain of wild animals is widely lacking, despite showing important differences in various immune parameters between the sexes (Kelly et al. 2018; Klein and Flanagan 2016; Valdebenito et al. 2021).

In the nervous system, immune function seems to be under particularly intense modulation since an insufficient response may result in infection, but an excessive response could result in prolonged inflammation and tissue damage (Kawli et al. 2010). Also, in tissues like the brain, general immune factors seem to serve a variety of non-immunological functions (Derecki et al. 2010; Radjavi et al. 2014). Furthermore, many variables may influence the immune response, including biotic and abiotic factors or the combination of both, as seen in some birds in the tropics that seem to upregulate aspects of their immune function in the wet season, presumably as defence mechanism against increased pathogen pressure that emerges from increased rainfall (Nwaogu et al. 2019; Tieleman et al. 2019).

The Kentish plover (*Charadrius alexandrinus*) is a small shorebird that is emerging as an ecological model system of sexually dimorphic reproduction and speciation (Székely 2019; Wang et al. 2019a, b). Kentish plovers are widely distributed along coastal and inland waterbodies across Eurasia and North Africa (del Hoyo et al. 2020). Previous studies on Kentish plover have found that males generally survive better than females in the wild (Eberhart-Phillips et al. 2018; Kosztolanyi et al. 2011). Though causes of sex-specific mortality may be non-exclusive and multifactorial (Székely et al. 2014), previous attempts in Kentish plover trying to link parasite burden and sex-specificity of infection have found comparable infection rates of blood parasites and pathogenic bacteria (Figuerola et al. 1996; Martínez-de la Puente et al. 2017; Valdebenito et al. 2020). Immune function, on the other hand, still remains undescribed in Kentish plover, being yet unknown whether males and females differ in immunity, or whether sex differences in immunity could be observed in the brain.

Here we investigate immune aspects in the male and female brain of Kentish plovers in two contrasting environments in China: the Bohai Bay located on the East coast and the Qinghai Lake located inland at high elevation in the Qinghai-Tibetan Plateau. We focus on expression patterns of immune system genes, since a healthy immune system in the brain is essential for protecting the animal against infection and maintaining cognitive ability (Gimsa et al. 2013; Marin and Kipnis 2013; Minias and Podlaszczuk 2017; Sol et al. 2007). We quantify the expression of genes annotated with immune functions in four brain regions, with the aim of evaluating sex differences in immune genes (Ham and Lee 2020). We test two specific hypotheses. First, based on sex differences in gene expression in the brain in various bird species (e.g. Delage and Cornil 2020; Rotllant et al. 2017; Sharma et al. 2014) and survival demographic analyses of Kentish plover (males survive better than females), we expect increased upregulation of immune genes in the male brain. Second, both Bohai Bay and the Qinghai Lake are stopovers of the East Asian-Australasian Flyway and the Central Asian Flyway, respectively. These environments show contrasting differences that could shape immune function due to local environmental factors such as pathogen pressure (e.g. Nwaogu et al. 2019). That is, increased chances of contact with other waterbirds, marine diversity, and their pathogens in the coastal site compared to the high-altitude inland site. Thus, we expect differences in immune gene expression between the sites, with a possible upregulation of immune genes in the coastal population.

## Results

### Differential expression analysis

**Fig. 1:**
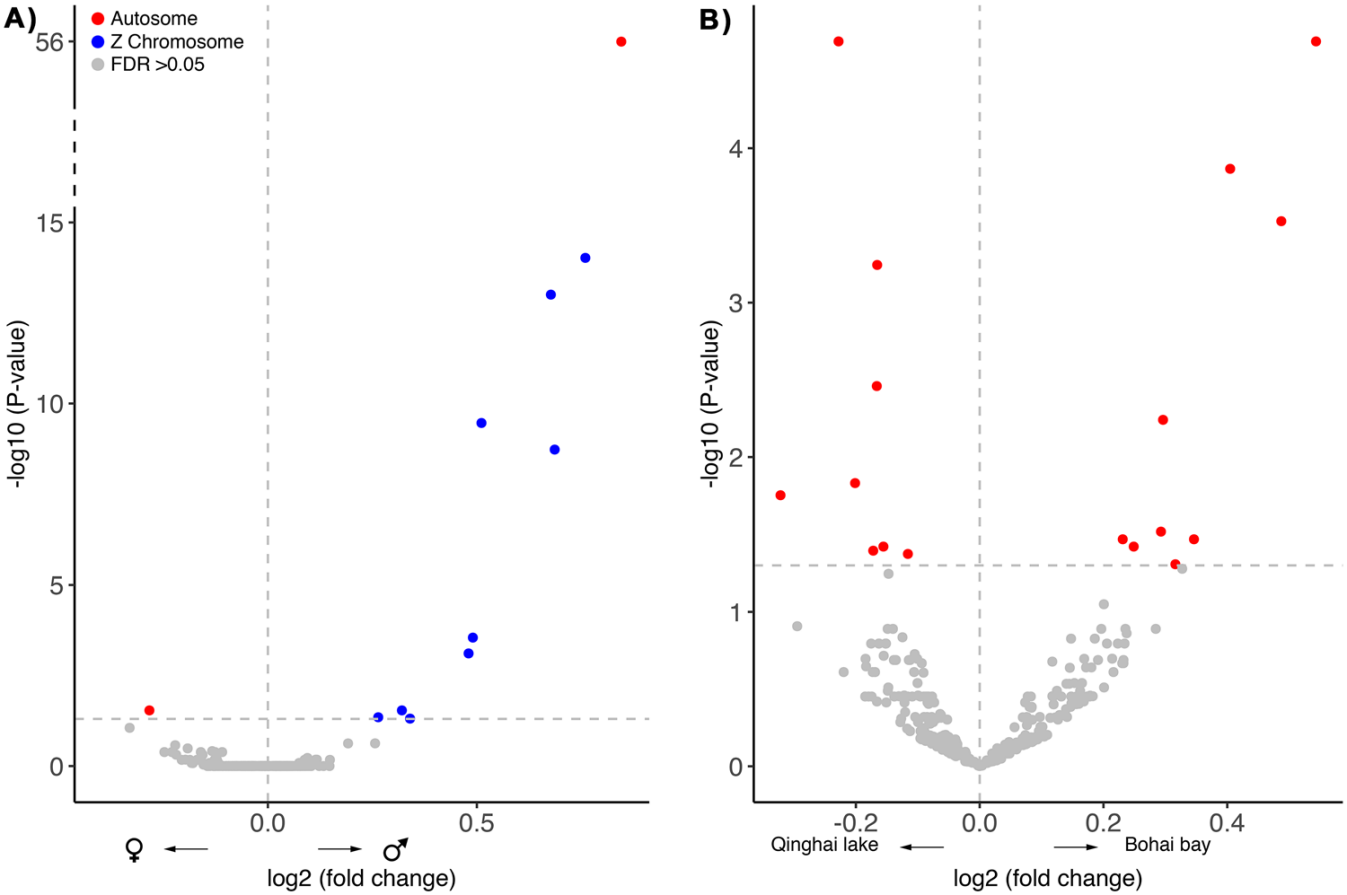
Biases in immune gene expression in brain tissue in Kentish plover. **A** Sex-specific immune gene expression where positive values indicate male-biased expression and negative a female bias. **B** Immune expression bias in relation to habitat. Colours indicate chromosomal location of the differentially expressed genes. The horizontal dashed line indicates a false discovery rate (FDR) threshold of 0.05

**Fig. 2:**
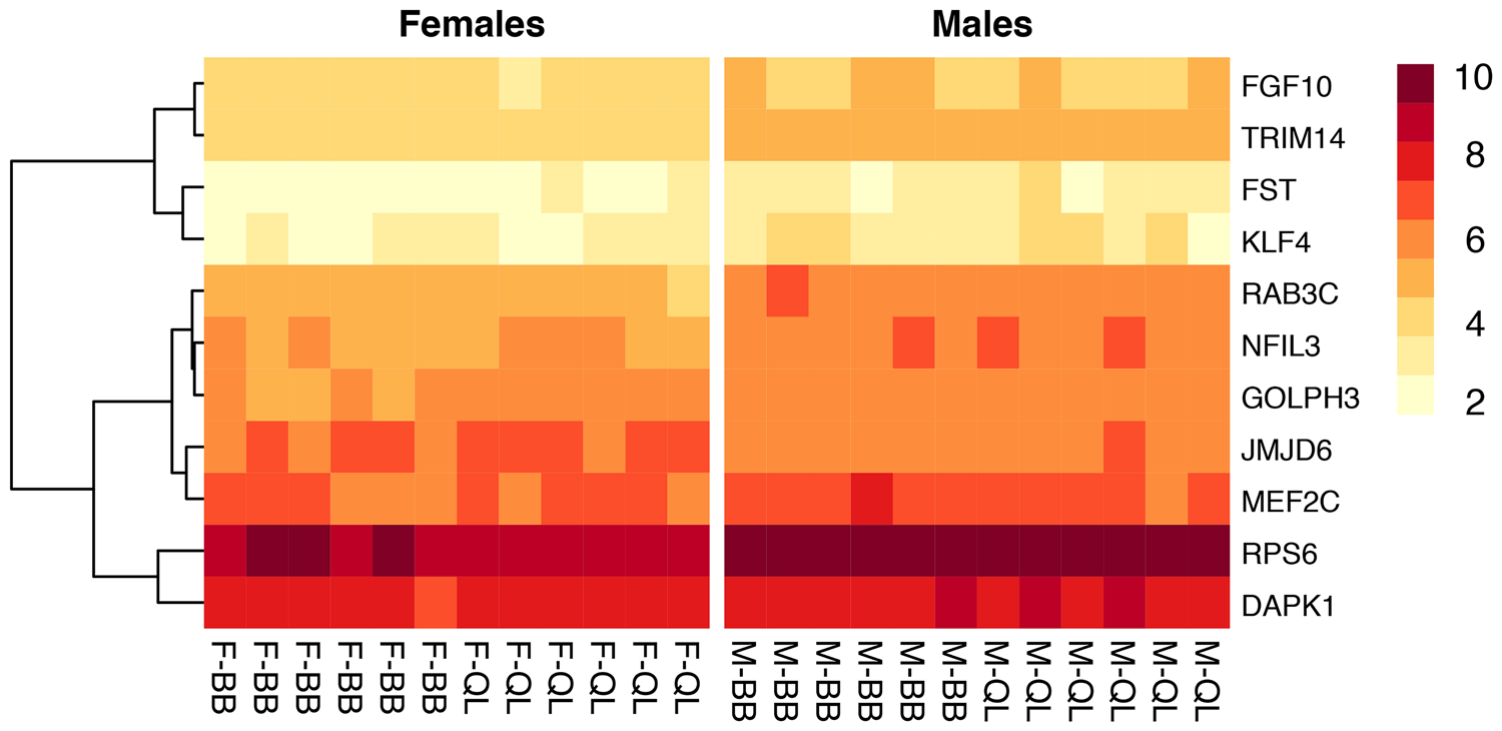
Heatmap of 11 significantly differentially expressed immune genes in 24 male and female Kentish plovers sampled in China. Colours represent normalised average expression counts (log2(*n* + 1)). Bottom column names refer to females (F) and males (M) from Bohai Bay (BB) and Qinghai Lake (QL)

We identified 403 transcripts associated with genes involved in immune processes (hereafter immune genes). After filtering out low counts (≤ 5), 379 immune genes were further processed. Of these, 11 genes had significant differences in expression pattern between the sexes, with 10 exhibiting male-biased upregulation and one having female-biased upregulation (Fisher’s exact test, *P*-value = 0.001, Table 1; Figs. 1A and 2). The male-biased immune genes were involved in the activation of various components of the immune system, whereas the female-biased gene was involved in T cell upregulation, consistent with a role in the activation of adaptive immunity (details in Table S1).

**Table 1:** Number of genes with biased expression according to **A) **sex, **B)** habitat, and **C)** their interaction in brain tissue of 24 Kentish plovers. The total number of genes investigated was 379. *P*-values refer to Fisher’s exact test of the difference in gene upregulation

**A**) Sex	Male-biased (upregulated)	Female-biased (upregulated)	*P*-value
	165 (10)	214 (1)	0.001
**B**) Habitat	Bohai Bay-biased (upregulated)	Qinghai Lake-biased (upregulated)	*P*-value
	186 (9)	193 (8)	0.807
**C**) Sex*habitat	Male-biased (upregulated)	Female-biased (upregulated)	*P*-value
Bohai Bay	180 (5)	199 (0)	0.024
Qinghai Lake	175 (5)	204 (0)	0.020

Comparing profiles of immune system gene expression between the two populations, we found 17 differentially expressed genes (Fig. 1B). The number of differentially upregulated immune genes did not differ between Bohai Bay and Qinghai Lake (9 and 8 genes, respectively; Fisher’s exact test, *P*-value = 0.807; Table 1). Here, gene functions corresponded largely to signal transduction associated to cytokine activation, but favouring functions of cellular metabolism in the Qinghai-Tibetan plateau (Table S2).

Interaction analysis showed a significant effect of sex on environment, where in both the coastal and inland location, the same five immune genes were significantly overexpressed in males but not in females (Table 1; Fig. S1).

### Chromosomal location

Out of 379 immune genes, 341 genes were located in the autosomes, 12 in the Z chromosome, with the remaining 26 genes not yet assigned to any chromosome. Nine out of 10 male-biased genes were linked to the Z chromosome, and one gene was located in the autosomes (Fig. 1). The location of the female-biased gene was in the autosomes (Fig. 1; Table 2a). In contrast, all differentially expressed genes identified when comparing the coastal and the inland environments (9 and 8 genes, respectively) were located in the autosomes (Fig. 1; Table 2b). Four out of 5 significantly male-biased overexpressed genes in the interaction analysis were located in the Z chromosome and one gene in the autosomes (Fig. S1).

**Table 2:** Genes differentially expressed by **A)** sex and **B)** habitat in brain tissue of 24 Kentish plovers from China

Gene name	Gene description	Upregulated	Chromosomal location
**A**) Sex			
JMJD6	Jumonji domain-containing 6	Females	Autosome
RPS6	Ribosomal protein S6	Males	Autosome
FGF10	Fibroblast growth factor 10	Males	Z chromosome
NFIL3	Nuclear factor, interleukin 3 regulated	Males	Z chromosome
GOLPH3	Golgi phosphoprotein 3	Males	Z chromosome
FST	Follistatin	Males	Z chromosome
TRIM14	Tripartite motif containing 14	Males	Z chromosome
KLF4	Kruppel like factor 4	Males	Z chromosome
MEF2C	Myocyte enhancer factor 2C	Males	Z chromosome
RAB3C	Ras-related protein Rab-3C	Males	Z chromosome
DAPK1	Death associated protein kinase 1	Males	Z chromosome
**B**) Habitat			
IGF1R	Insulin like growth factor 1 receptor	Bohai Bay	Autosome
BRAF	B-Raf proto-oncogene, serine/threonine kinase	Bohai Bay	Autosome
FOXO3	Forkhead Box O3	Bohai Bay	Autosome
CHAAL00000005676*		Bohai Bay	Autosome
NRARP	NOTCH regulated ankyrin repeat protein	Bohai Bay	Autosome
EGR1	Early growth response 1	Bohai Bay	Autosome
FOXP1	Forkhead box P1	Bohai Bay	Autosome
SFRP1	Secreted frizzled related protein 1	Bohai Bay	Autosome
IGFBP2	Insulin like growth factor binding protein 2	Bohai Bay	Autosome
ACP6	Acid phosphatase 6	Qinghai Lake	Autosome
PRDX3	Peroxiredoxin 3	Qinghai Lake	Autosome
P4HTM	Prolyl 4-hydroxylase, transmembrane	Qinghai Lake	Autosome
LOC105410688		Qinghai Lake	Autosome
IMPDH2	Inosine monophosphate dehydrogenase 2	Qinghai Lake	Autosome
GPI	Glucose-6-phosphate isomerase	Qinghai Lake	Autosome
CTNNB1	Catenin beta 1	Qinghai Lake	Autosome
ATP1B1	ATPase Na + /K + transporting subunit beta 1	Qinghai Lake	Autosome

*No gene name assigned

## Discussion

Here we showed that the vast majority of immune genes have similar expression levels between males and females, and between two ecologically different habitats. We identified 10 immune genes which had higher expression in males and one gene that was more highly expressed in females, though these represent only ~ 2.9% of all genes in the analysis. We further note that the magnitude of the differential gene expression was small, in all cases no larger than − 1/ + 1 Log2 fold change (full details in Table S3–S6).

Recent studies are addressing specific aspects of immune defence using transcriptomes in birds (e.g. Scalf et al. 2019; Videvall et al. 2015, 2020). Despite the growing importance of sex-specific research across disciplines (Moghadam et al. 2013; Wilson 2020), only the work of Wang et al. (2019c) has so far explored sex differences using blood samples in captive Eurasian magpies (*Pica pica*), finding important sex-biases in expression of genes related to stress resistance, immunity, energy metabolism, reproduction, and lifespan regulation. Genomic methods are powerful since they introduce a more holistic perspective in regards to a functional gene group, but it should be noted that the correlation between mRNA and protein concentrations could be inconsistent (Li et al. 2014; Lu et al. 2007) and that tissue type may influence gene expression profiles (Watson et al. 2017).

Differential gene expression between the male and female brain has been demonstrated in several species, but an emphasis on immune genes is rarely seen, hence the little knowledge about possible causes and consequences of this sex difference. Studies not distinguishing for sex in murine models describe an association between increased activation of innate immune genes in the brain and neuronal aging (Cribbs et al. 2012), alterations of social behaviour (Ma et al. 2015), and the risk of developing schizophrenia (Comer et al. 2020). Note that the microglia, the major source of immune genes in the brain, is known to regulate the brain functional connectivity and behaviour (Chen et al. 2010; Derecki et al. 2012; Squarzoni et al. 2014; Zhan et al. 2014). Moreover, genes associated with, for example, the major histocompatibility complex (MHC), the complement, and their receptors, are known to be expressed in the brain and regulate brain structural and functional plasticity, either directly or indirectly by controlling microglial or immune activation (McAllister 2014; Perry and O'Connor 2008; Tian et al. 2009). Perhaps, sex-specific effects could be linked to the sex hormones, since the main female sex hormone, oestrogen, has been associated with several roles in the microglia, thought to drive behavioural differences during neuronal development in mammalian and avian models (Delage and Cornil 2020; Martin-Jiménez et al. 2019; Siani et al. 2017; Yun et al. 2018). Importantly, our results showed a male bias in highly differentially expressed immune genes. This coincides with our expectations based on demographic studies (biases in mortality and adult sex ratio; Eberhart-Phillips et al. 2018; Kosztolanyi et al. 2011), but further studies are needed to explore whether our findings in the brain actually entail a mortality cost for this bird. Though, perhaps in specific infections such as the filarial nematodes *Chandlerella quiscali* that often invades brain tissue in wild birds (Atkinson et al. 2008; Edwards et al. 2017), the difference in immune gene expression between males and females could determine a sex-specific advantage in defence. Also, it has been shown that intraspecific variation of the microglia between the sexes is of common occurrence in several neurodegenerative diseases in mammals (Martin-Jiménez et al. 2019; Schwarz et al. 2012; Villa et al. 2018); thus, our findings could be potentially linked with the development of such diseases in this manner. Unfortunately, research on sex-specific neurodegenerative diseases in wild birds is in its infancy.

Differentially expressed genes in males largely included roles in immune defence, control of cellular proliferation, and cytokine mediators. Only half of these genes were associated to roles in neuronal pathways, which may suggest that these immune genes might have a moderate to minor role in specific neuronal functions, possibly because, though unlikely, residual blood could remain trapped within the brain tissue. Our results showed that upregulated immune genes in the brain in the coastal environment was similar to the high-altitude habitat in the Qinghai-Tibetan plateau. Because many immune genes expressed in the brain are believed to regulate behaviour and other cellular functions, this suggests that, in the individuals sampled, the genetic control of these processes seems rather uniform between environments. Furthermore, though our findings could also suggest that pathogen pressure in both environments were comparable (Lindström et al. 2004; Peuß et al. 2020), we deem this unlikely since overexpressed genes in the coastal environment seemed to largely serve functions of signal transduction posterior to cytokine activation (e.g. the FOXO3 and FOXP1 genes), whereas in the Qinghai-Tibetan Plateau, there was more activation of genes associated to cellular metabolism, possibly due to the extreme environmental conditions such as high altitude. Interestingly, males from both the coastal and high-altitude environments had an overexpression of the same five genes, serving general functions of immune cell activation, with no evidence of activation of genes related to metabolism. One interpretation of this is that males of this species could have a similar baseline immune activation across the two environments.

We found that most male-biased genes were located on the Z chromosome. Interestingly, when comparing differential immune gene expression between the two environments, all upregulated genes belonged to the autosomes. This could suggest that the sex-biases in immune gene expression in Kentish plover are linked to the sex chromosomes, possibly expected due to the absence of dosage compensation in most birds (Heard and Disteche 2006; Itoh et al. 2007; Parsch and Ellegren 2013; Zhou et al. 2014). Unfortunately, there is not yet an assembly available for the W chromosome in Kentish plover (Wang et al. 2019a), which truncates further conclusions. Wang et al. (2019c) found in Eurasian magpies that, in general, males show more upregulated genes than females in the blood, although some specific immune pathways were found to be upregulated in females. Unfortunately, Wang et al. (2019c) did not characterise the chromosomal location of these genes.

## Conclusion

The use of genomic methods is a convenient approach for addressing a wide range of specific questions. Though caution at interpretation is required because often single genes have several biological functions and these can differ between tissue type. Here we showed that, though small, sex but not environment has an effect on the upregulation of immune gene expression in Kentish plover. We do not know the impact that such differences could have on the bird’s life trajectory, but we nevertheless note that the direction of the sex-bias in immune genes met the expected predictions based on higher survival rate of male Kentish plover over females in the wild (Eberhart-Phillips et al. 2018). We are aware, however, that sex-specific mortality can be multifactorial and future studies should further the knowledge of sex differences in immune gene expression in this shorebird.

## Materials and methods

### Sampling

Fieldwork took place at two sites in China where the Kentish plover is a locally abundant breeder (Fig. S2): Bohai Bay at sea level (39° 7′6.22"N, 118°11′49.84"E; more details of the site are described in Que et al. 2015), and Qinghai Lake (Koko Nor), the largest lake in China at 3200 m above sea level (36°45′56.86"N, 100°43′21.68"E; Jian et al. 2021). Fieldwork was conducted during the breeding period in May 2015, capturing 24 incubating adults on the nest (6 males and 6 females at each site) followed by morphometric measurements according to a standard protocol (see Székely et al. 2008). To obtain blood and brain tissues, the birds were sacrificed conforming to the regulations of ethical conditions by the Chinese Animal Welfare Act (20,090,606), and of the Animal Welfare and Ethical Review Body of the University of Bath and the Institutional Ethical Committee of Animal Experimentation of Sun Yat-sen University (2005DKA21403-JK). This study did not involve endangered or protected species.

Tissue samples were collected from four brain regions based on their spatial location (basal forebrain and brainstem): the hypothalamus, medial extended amygdala, nucleus accumbens, and septum (Fig. S3). Apart from their specific function mediating social behaviour, these regions are run through several axonal tracts from superior areas in the forebrain.

Using a stainless-steel rat brain matrix, brain samples were dissected by first cutting the brain coronally in order to rostrocaudally standardise the tissue slabs, then each region of interest was manually dissected using a portable dissection microscope following a chicken brain atlas (Puelles et al. 2018). The Kentish plover and the chicken brain differ in morphology; thus, we used visible anatomical landmarks to define the approximate margins of the dissections. Brain tissue samples were stored in RNAlater (Qiagen) and kept cold (in the field) using either liquid nitrogen or cold blocks. At the end of each day, samples were stored in freezers (− 20 °C) and then once at the lab samples were stored at − 80 °C prior RNA extraction.

### RNA extraction and sequencing

RNA extraction and sequencing of 96 brain samples were performed by Novogene Beijing. RNA degradation and contamination were monitored using 1% agarose gels, and RNA purity was assessed using a NanoPhotometer spectrometer (IMPLEN, CA, USA). RNA concentration and integrity were measured using a Qubit 2.0 Flurometer (Life Technologies, Carlsbad, CA, USA) and an Agilent Bioanalyzer 2100, respectively. Sequencing libraries preparation used 3 μg RNA per sample and cluster formation was conducted following manufacturer’s recommendations (TruSeq PE Cluster Kit v3-cBot-HS, Illumina). Sequencing was performed on an Illumina HiSeq platform. The outcome generated paired-end reads with a fragment length of 300–500 bp and an average paired end length of 150 bp, for a total of 519 millions of raw paired-end reads sequenced (~ 5.4 million paired-end reads per sample).

### Transcriptome profile annotation

Reads were cleaned using Trimmomatic v0.35. All further analysis used these high-quality reads. Cleaned reads were aligned to the Kentish plover genome (Wang et al. (2019a); assembly ID ASM871129v1) using HISAT2 v2.1.0 (Kim et al. 2013). We used Samtools v1.2 (Li et al. 2009) to clean HISAT2 outputs in order to remove multi-mapped reads, unmapped reads, and unmapped mates (-q 40, -f 2, -F 12). Counts of raw read were extracted from cleaned HISAT2 outputs using HTSeq-count v0.11.2 (Anders et al. 2015).

### Differential expression analysis

Differential expression analysis was performed on two different groupings of the dataset, i.e. population (Bohai Bay *versus* Qinghai Lake), sex, and the two-way interaction of population and sex. Genes were filtered and removed if the sum of all 24 samples had 5 or less counts per million mapped reads.

To examine expression performance of the regions sampled, we conducted a principal component analysis (PCA) that revealed no major distinction in expression between the regions except for the nucleus accumbens (Fig. S3). Importantly, the PCA suggests consistent tissue sampling in the field. Thus, we pooled all brain regions together (averaging counts of the four regions) for further analysis. The analysis of differential gene expression was performed between the sexes and populations using the function *DESeq* from the R statistical package *DeSeq2* (Love et al. 2014). This function calculates differential expression based on the negative binomial (i.e. Gamma-Poisson) distribution, which is an advantage over earlier binomial models because it makes fewer type I errors (Anders and Huber 2010). The function operates estimating size factors, then estimating dispersion of each gene, to finish fitting negative binomial generalised linear models and Wald statistics (see *DESeq* documentation for further details). *DESeq* results were extracted specifying contrasts (male *versus* females; Bohai Bay *versus* Qinghai Lake) and false discovery rate < 0.05. Analyses were conducted in R version 3.3.2 (R Core Team 2016).

### Gene ontology annotations and set enrichment analyses

Gene ontology enrichment categories and the extraction of GO terms followed methods previously described in Wang et al. (2019a). Briefly, we used BLASTP v.3.2.0 + (with an E-value of 1e-5 Altschul et al. 1990) to BLAST Kentish plover proteins against the RefSeq protein database. GO terms were then assigned using Blast2GO software v.4.1.9 (Götz et al. 2008) and merged with GO terms obtained from InterProScan v.5.25 (parameters: -f xml, -goterms, -iprlookup) (Jones et al. 2014). We evaluated GO terms from the domain biological processes and then the subcategory immune system processes. The gene enrichment analyses were performed using a hypergeometric test for overrepresentation and corrected for false positives with the Benjamini and Hochberg FDR correction (Benjamini and Hochberg 1995). Functional groups with a FDR *P*-value < 0.05 were regarded as statistically significantly overrepresented. Cases where the expected number of genes less than one were only classified as significantly enriched if the observed number of genes was higher than one.

### Sex- and population-specific gene expression

We used Fisher’s exact tests to examine for differences between males and females in the proportion of up and downregulated sex-biased genes. The same method was used in statistical comparisons between population, and in the sex × population interaction.

## Supplementary Information

Below is the link to the electronic supplementary material.

Supplementary file1 (PDF 1482 KB)

Supplementary file2 (XLSX 167 KB)

## Data Availability

The full dataset can be downloaded from https://figshare.com/s/153d63db7e798269aa5d. Genome assembly is available at NCBI under accession ID ASM871129v1.
