# Supplementary material for: Sex differences in immune gene expression in the brain of a small shorebird
Source: Immunogenetics. 2022 Jan 27;74(5):487–96. doi: 10.1007/s00251-022-01253-w (PMC8792134; doi:10.1007/s00251-022-01253-w)
Supplement: Supplementary file 1 — Supplementary file1 (PDF 1482 KB) [file 251_2022_1253_MOESM1_ESM.pdf]

Supplementary material:

Sex differences in immune gene expression in the brain of a small shorebird

José O. Valdebenito[1,2], Kathryn H. Maher[1,3,4], Gergely Zachár[5], Qin Huang[6], Zhengwang Zhang[4], Larry J. Young[7], Tamás Székely[1,2,6], Pinjia Que[4], Yang Liu[6], Araxi O. Urrutia[1,8]

[1]*Milner Centre for Evolution, Department of Biology and Biochemistry, University of Bath, Bath, United Kingdom*

[2]*Department of Evolutionary Zoology and Human Biology, University of Debrecen, Debrecen, Hungary*

[3]*Department of Animal and Plant Sciences, University of Sheffield, Sheffield, United Kingdom*

[4]*Ministry of Education Key Laboratory for Biodiversity Sciences and Ecological Engineering, College of Life Sciences, Beijing Normal University, Beijing, China*

[5]*Department of Anatomy, Histology and Embryology, Semmelweis University, Budapest, Hungary*

[6]*State Key Laboratory of Biocontrol, School of Ecology, Sun Yat-sen University, Guangzhou, China*

[7]*Silvio O. Conte Center for Oxytocin and Social Cognition, Center for Translational Social Neuroscience, Department of Psychiatry and Behavioral Sciences, Yerkes National Primate Research Center, Emory University, Atlanta, USA*

[8]*Instituto de Ecologia, Universidad Nacional Autonoma de México, Ciudad de México, México*

Correspondence: José O. Valdebenito, j.valdebenito.ch@gmail.com

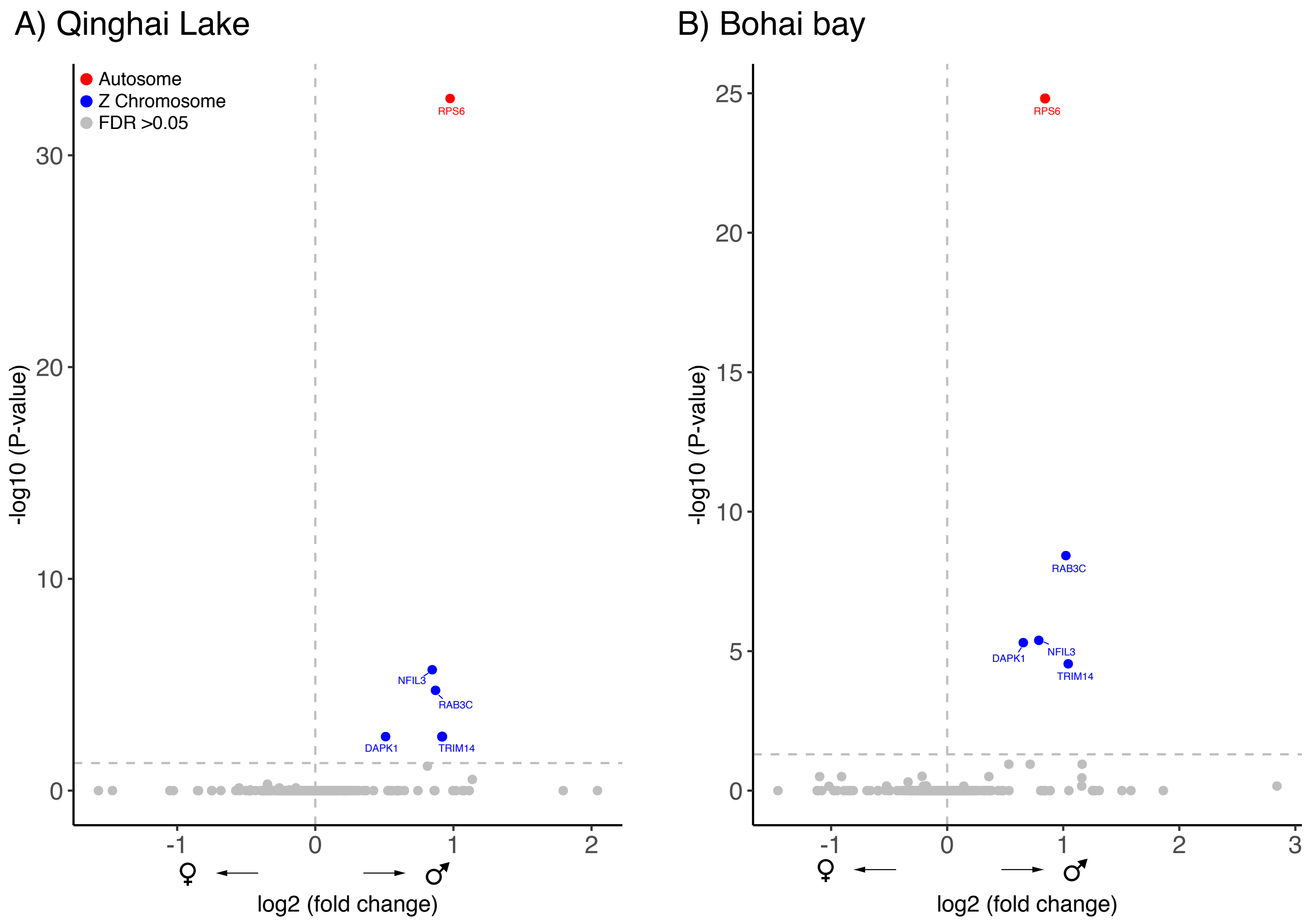

Fig. S1 Differential expression of sex-biased immune genes in Qinghai Lake and Bohai Bay, China. Colours indicate chromosomal location of the differentially expressed genes. The horizontal dashed line indicates a false discovery rate (FDR) threshold of 0.05. Note that in both locations the same genes are overexpressed

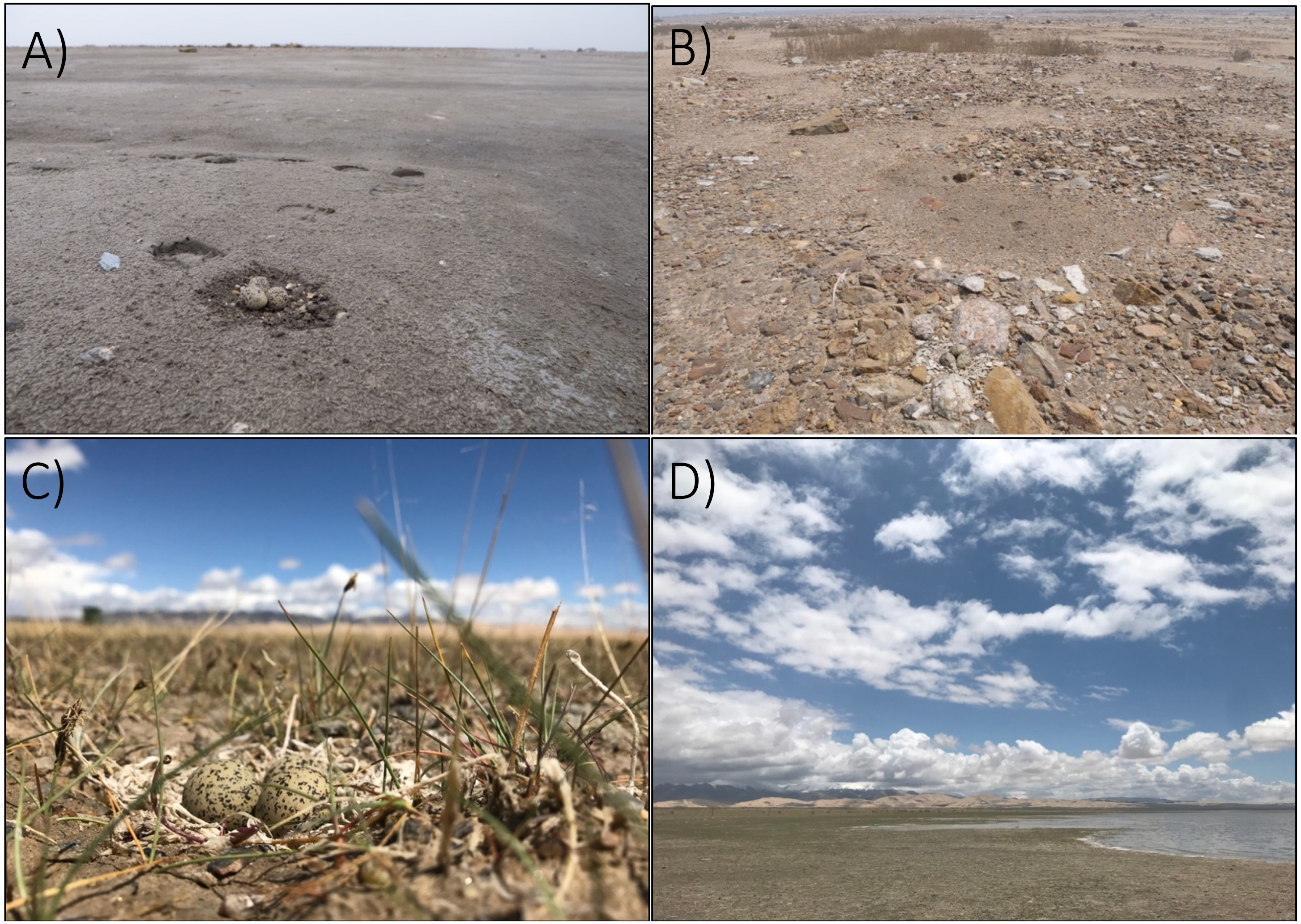

Fig. S2 Comparison of the two environments studied. A) and B) depict two nests at Bohai Bay. C) shows a nest and D) a panoramic view of the steppe landscape at Qinghai Lake

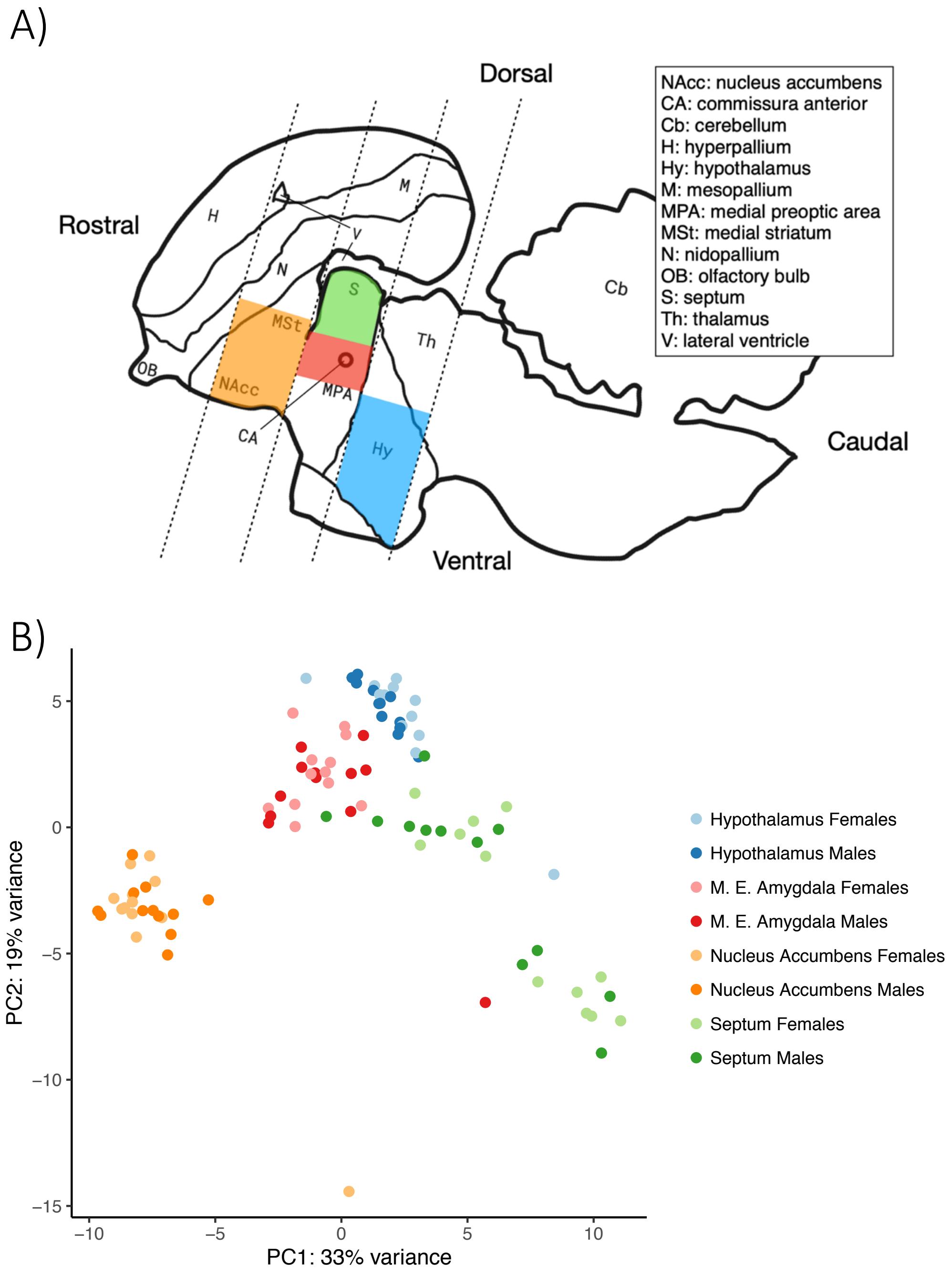

Fig. S3 Four regions in the brain of Kentish plover that were sampled in the present study. Note that all regions are in close proximity to each other. A) Neuroanatomy indicating approximate location of the four regions and B) principal components analysis of those regions in relation to sex. M. E. Amygdala = medial extended amygdala

Table S1 Genes upregulated by sex in Kentish plover and its function according to Gene Ontology (GO)

Gene ID	Gene symbol	Gene description	Sex upregulated	Chromosomal location	GO biological functions
CHAAL00000003424	JMJD6	Jumonji Domain Containing 6, Arginine Demethylase And Lysine Hydroxylase	Females	Autosome	-Macrophage activation<br>-T-cell differentiation in thymus<br>-Erythrocyte development
CHAAL00000001967	RPS6	Ribosomal Protein S6	Males	Autosome	-Activation-induced cell death of T cells<br>-Negative regulation of apoptotic process<br>-Positive regulation of apoptotic process<br>-T-cell differentiation in thymus<br>-T-cell proliferation involved in immune response<br>-TOR signalling<br>-Viral transcription<br>-Erythrocyte development<br>-G1/S transition of mitotic cell cycle<br>-Gastrulation<br>-Glucose homeostasis<br>-Mitotic cell cycle checkpoint<br>-Mitotic nuclear division<br>-Nuclear-transcribed mRNA catabolic process<br>-Nonsense-mediated decay<br>-Oogenesis stage<br>-Ribosomal small subunit biogenesis<br>-rRNA processing<br>-Placenta development<br>-Translational initiation<br>-SRP-dependent co-translational protein targeting to membrane
CHAAL00000002488	FGF10	Fibroblast Growth Factor 10	Males	Z chromosome	-Positive regulation of lymphocyte proliferation<br>-Thymus development<br>-Spleen development
CHAAL00000002733	NFIL3	Nuclear Factor, Interleukin 3 Regulated	Males	Z chromosome	-Immune response<br>-Regulation of transcription, DNA-templated<br>-Transcription from RNA polymerase II promoter<br>-Circadian rhythm
CHAAL00000007024	GOLPH3	Golgi Phosphoprotein 3	Males	Z chromosome	-Leukocyte tethering or rolling

CHAAL00000007046	FST	Follistatin	Males	Z chromosome	-Haematopoietic progenitor cell differentiation
CHAAL00000012274	TRIM14	Tripartite Motif Containing 14	Males	Z chromosome	-Innate immune response<br>-Negative regulation of viral transcription<br>-Positive regulation of NF-kappaB transcription factor activity
CHAAL00000012457	KLF4	Kruppel Like Factor 4	Males	Z chromosome	-Epidermal cell differentiation<br>-Negative regulation of cell proliferation<br>-Negative regulation of cysteine-type endopeptidase activity involved in apoptotic process<br>-Negative regulation of heterotypic cell-cell adhesion<br>-Negative regulation of interleukin-8 biosynthetic process<br>-Negative regulation of NF-kappaB transcription factor activity<br>-Negative regulation of response to cytokine stimulus<br>-Positive regulation of nitric oxide biosynthetic process<br>-Positive regulation of transcription regulatory region DNA binding<br>-Stem cell population maintenance<br>-Canonical Wnt signalilng pathway<br>-Cellular response to growth factor stimulus<br>-Cellular response to laminar fluid shear stress<br>-Epidermis morphogenesis<br>-Fat cell differentiation<br>-Negative regulation of cell migration involved in sprouting angiogenesis<br>-Negative regulation of chemokine (C-X-C motif) ligand 2 production<br>-Negative regulation of transcription from RNA polymerase II promoter<br>-Positive regulation of cellular protein metabolic process<br>-Positive regulation of haemoglobin biosynthetic process<br>-Positive regulation of telomerase activity<br>-Positive regulation of transcription from RNA polymerase II promoter<br>-Post-embryonic camera-type eye development<br>-Post-embryonic haemopoiesis<br>-Regulation of axon regeneration<br>-Response to retinoic acid

					-Transcription from RNA polymerase II promoter
CHAAL00000012612	MEF2C	Myocyte Enhancer Factor 2C	Males	Z chromosome	-B-cell homeostasis<br>-B-cell proliferation<br>-B-cell receptor signalling pathway<br>-Germinal centre formation<br>-Humoral immune response<br>-Monocyte differentiation<br>-Platelet formation<br>-Positive regulation of B-cell proliferation<br>-Regulation of germinal centre formation<br>-Regulation of megakaryocyte differentiation
CHAAL00000013158	RAB3C	Ras-related protein Rab-3C	Males	Z chromosome	-Antigen processing and presentation
CHAAL00000014271	DAPK1	Death Associated Protein Kinase 1	Males	Z chromosome	-Cellular response to interferon-gamma<br>-Extrinsic apoptotic signalling pathway via death domain receptors<br>-Intracellular signal transduction<br>-Negative regulation of extrinsic apoptotic signalling pathway via death domain receptors<br>-Negative regulation of translation<br>-Positive regulation of cysteine-type endopeptidase activity involved in apoptotic process<br>-Protein autophosphorylation<br>-Regulation of N-methyl-D-aspartate selective glutamate receptor activity

Table S2 Genes upregulated by environment in Kentish plover and their function according to Gene Ontology (GO)

Gene ID	Gene symbol	Gene description	Environment upregulated	Chromosomal location	GO biological functions
CHAAL00000000238	IGF1R	Insulin Like Growth Factor 1 Receptor	Bohai Bay: Coastal	Autosomes	– immune response<br>– inactivation of MAPKK activity<br>– insulin-like growth factor receptor signaling pathway<br>– negative regulation of apoptotic process<br>– peptidyl-tyrosine autophosphorylation<br>– phosphatidylinositol-mediated signaling<br>– positive regulation of cell migration<br>– positive regulation of DNA replication<br>– protein tetramerization<br>– regulation of JNK cascade
CHAAL00000001793	ACP6	Acid Phosphatase 6, Lysophosphatidic	Qinghai Lake: Tibetan plateau	Autosomes	– Dephosphorylation<br>– hematopoietic progenitor cell differentiation
CHAAL00000002897	BRAF	B-Raf Proto-Oncogene, Serine/Threonine Kinase	Bohai Bay: Coastal	Autosomes	– CD4-positive, alpha-beta T cell differentiation<br>– cellular response to calcium ion<br>– cellular response to drug<br>– establishment of protein localization to membrane<br>– face development<br>– glucose transport<br>– head morphogenesis<br>– intracellular signal transduction<br>– long-term synaptic potentiation<br>– myeloid progenitor cell differentiation<br>– negative regulation of endothelial cell apoptotic process<br>– negative regulation of fibroblast migration<br>– negative regulation of neuron apoptotic process<br>– negative regulation of synaptic vesicle exocytosis<br>– positive regulation of axon regeneration<br>– positive regulation of axonogenesis<br>– positive regulation of ERK1 and ERK2 cascade<br>– positive regulation of gene expression<br>– positive regulation of peptidyl-serine phosphorylation<br>– positive regulation of stress fiber assembly<br>– positive regulation of substrate adhesion-dependent cell spreading<br>– positive T cell selection<br>– protein phosphorylation<br>– regulation of cell proliferation<br>– somatic stem cell population maintenance

					– thymus development<br>– thyroid gland development<br>– visual learning
CHAAL00000004570	PRDX3	Peroxiredoxin 3	Qinghai Lake: Tibetan plateau	Autosomes	– apoptotic process<br>– cellular oxidant detoxification<br>– cellular response to reactive oxygen species<br>– hydrogen peroxide catabolic process<br>– mitochondrion organization<br>– myeloid cell differentiation<br>– negative regulation of cysteine-type endopeptidase activity involved in apoptotic process<br>– negative regulation of kinase activity<br>– peptidyl-cysteine oxidation<br>– positive regulation of cell proliferation<br>– positive regulation of NF-kappaB transcription factor activity<br>– regulation of mitochondrial membrane potential<br>– response to hydrogen peroxide<br>– response to lipopolysaccharide
CHAAL00000004722	FOXO3	Forkhead Box O3	Bohai Bay: Coastal	Autosomes	– antral ovarian follicle growth<br>– cellular response to oxidative stress<br>– DNA damage response, signal transduction by p53 class mediator<br>– extrinsic apoptotic signaling pathway in absence of ligand<br>– glucose homeostasis<br>– initiation of primordial ovarian follicle growth<br>– negative regulation of canonical Wnt signaling pathway<br>– negative regulation of transcription from RNA polymerase II promoter<br>– neuronal stem cell population maintenance<br>– oocyte maturation<br>– ovulation from ovarian follicle<br>– positive regulation of erythrocyte differentiation<br>– positive regulation of neuron apoptotic process<br>– positive regulation of transcription from RNA polymerase II promoter<br>– regulation of neural precursor cell proliferation<br>– regulation of reactive oxygen species metabolic process<br>– regulation of translation<br>– transcription from RNA polymerase II promoter<br>– tumor necrosis factor-mediated signaling pathway
CHAAL00000005676	NA		Bohai Bay: Coastal	Autosomes	– adrenal gland development<br>– anterior/posterior pattern specification<br>– branching involved in ureteric bud morphogenesis

					– embryonic hemopoiesis<br>– embryonic limb morphogenesis<br>– embryonic skeletal system development<br>– negative regulation of neuron differentiation<br>– negative regulation of sequence-specific DNA binding transcription factor activity<br>– positive regulation of cell proliferation<br>– positive regulation of G2/M transition of mitotic cell cycle<br>– positive regulation of transcription from RNA polymerase II promoter<br>– proximal/distal pattern formation<br>– regulation of ossification<br>– spleen development<br>– thymus development<br>– transcription from RNA polymerase II promoter
CHAAL00000008647	NRARP	NOTCH Regulated Ankyrin Repeat Protein	Bohai Bay: Coastal	Autosomes	– blood vessel endothelial cell proliferation involved in sprouting angiogenesis<br>– negative regulation of Notch signaling pathway involved in somitogenesis<br>– negative regulation of T cell differentiation<br>– negative regulation of transcription from RNA polymerase II promoter<br>– Notch signaling pathway<br>– patterning of blood vessels<br>– positive regulation of canonical Wnt signaling pathway<br>– positive regulation of endothelial cell proliferation<br>– somite rostral/caudal axis specification
CHAAL00000008726	P4HTM	Prolyl 4-Hydroxylase, Transmembrane	Qinghai Lake: Tibetan plateau	Autosomes	– oxidation-reduction process<br>– regulation of erythrocyte differentiation
CHAAL00000009009	EGR1	Early Growth Response 1	Bohai Bay: Coastal	Autosomes	– BMP signaling pathway<br>– cellular response to gamma radiation<br>– interleukin-1-mediated signaling pathway<br>– motor learning<br>– negative regulation of canonical Wnt signaling pathway<br>– negative regulation of transcription from RNA polymerase II promoter<br>– positive regulation of transcription from RNA polymerase II promoter<br>– regulation of apoptotic process<br>– regulation of protein sumoylation<br>– regulation of transcription from RNA polymerase II promoter in response to hypoxia

					– response to auditory stimulus<br>– response to glucose<br>– response to insulin<br>– skeletal muscle cell differentiation<br>– T cell differentiation<br>– transcription from RNA polymerase II promoter
CHAAL00000010987	LOC105410688		Qinghai Lake: Tibetan plateau	Autosomes	– cell aging<br>– cell proliferation<br>– cell surface receptor signaling pathway<br>– DNA damage response, signal transduction by p53 class mediator<br>– histone acetylation<br>– inflammatory response<br>– innate immune response<br>– negative regulation of cell aging<br>– negative regulation of cell cycle arrest<br>– negative regulation of cellular protein metabolic process<br>– negative regulation of DNA damage response, signal transduction by p53 class mediator<br>– negative regulation of gene expression<br>– negative regulation of intrinsic apoptotic signaling pathway in response to DNA damage by p53 class mediator<br>– negative regulation of mature B cell apoptotic process<br>– negative regulation of myeloid cell apoptotic process<br>– positive chemotaxis<br>– positive regulation of arachidonic acid secretion<br>– positive regulation of B cell proliferation<br>– positive regulation of chemokine (C-X-C motif) ligand 2 production<br>– positive regulation of cytokine secretion<br>– positive regulation of ERK1 and ERK2 cascade<br>– positive regulation of fibroblast proliferation<br>– positive regulation of lipopolysaccharide-mediated signaling pathway<br>– positive regulation of MAP kinase activity<br>– positive regulation of myeloid leukocyte cytokine production involved in immune response<br>– positive regulation of peptidyl-serine phosphorylation<br>– positive regulation of peptidyl-tyrosine phosphorylation<br>– positive regulation of prostaglandin secretion involved in immune response<br>– positive regulation of protein kinase A signaling<br>– prostaglandin biosynthetic process

					− protein homotrimerization<br>− regulation of transcription, DNA-templated
CHAAL00000012589	LOC101870 525	Inosine Monophosphate Dehydrogenase 2	Qinghai Lake: Tibetan plateau	Autosomes	− cellular response to interleukin-4<br>− GMP biosynthetic process<br>− lymphocyte proliferation<br>− oxidation-reduction process
CHAAL00000013053	GPI	Glucose-6-Phosphate Isomerase	Qinghai Lake: Tibetan plateau	Autosomes	− erythrocyte homeostasis<br>− gluconeogenesis<br>− glucose homeostasis<br>− glycolytic process<br>− mesoderm formation
CHAAL00000014900	FOXP1	Forkhead Box P1	Bohai Bay: Coastal	Autosomes	− cardiac muscle cell differentiation<br>− cardiovascular system development<br>− immunoglobulin V(D)J recombination<br>− interleukin-21 secretion<br>− lung secretory cell differentiation<br>− motor neuron axon guidance<br>− negative regulation of lung goblet cell differentiation<br>− negative regulation of transcription from RNA polymerase II promoter<br>− positive regulation of cardiac muscle cell differentiation<br>− positive regulation of epithelial cell proliferation<br>− positive regulation of immunoglobulin production<br>− positive regulation of mesenchymal cell proliferation<br>− positive regulation of transcription from RNA polymerase II promoter<br>− pre-B cell differentiation<br>− regulation of cardiac muscle cell proliferation<br>− sarcomere organization<br>− skeletal muscle tissue development<br>− smooth muscle tissue development<br>− T follicular helper cell differentiation<br>− transcription, DNA-templated<br>− ventral spinal cord development

CHAAL00000014682	SFRP1	Secreted Frizzled Related Protein 1	Bohai Bay: Coastal	Autosomes	bone trabecula formation<br>canonical Wnt signaling pathway<br>cellular response to BMP stimulus<br>cellular response to estradiol stimulus<br>cellular response to estrogen stimulus<br>cellular response to fibroblast growth factor stimulus<br>cellular response to heparin<br>cellular response to hypoxia<br>cellular response to interleukin-1<br>cellular response to prostaglandin E stimulus<br>cellular response to starvation<br>cellular response to transforming growth factor beta stimulus<br>cellular response to tumor necrosis factor<br>cellular response to vitamin D<br>cellular response to X-ray<br>convergent extension involved in somitogenesis<br>digestive tract morphogenesis<br>dopaminergic neuron differentiation<br>dorsal/ventral axis specification<br>female gonad development<br>hematopoietic stem cell differentiation<br>male gonad development<br>midbrain morphogenesis<br>negative regulation of androgen receptor signaling pathway<br>negative regulation of B cell differentiation<br>negative regulation of BMP signaling pathway<br>negative regulation of bone remodeling<br>negative regulation of canonical Wnt signaling pathway involved in controlling type B pancreatic cell proliferation<br>negative regulation of cell growth<br>negative regulation of cell migration<br>negative regulation of epithelial cell proliferation<br>negative regulation of epithelial to mesenchymal transition<br>negative regulation of fibroblast apoptotic process<br>negative regulation of fibroblast proliferation<br>negative regulation of insulin secretion<br>negative regulation of JUN kinase activity<br>negative regulation of osteoblast differentiation<br>negative regulation of osteoblast proliferation<br>negative regulation of osteoclast differentiation<br>negative regulation of peptidyl-tyrosine phosphorylation<br>negative regulation of planar cell polarity pathway involved in axis elongation

					negative regulation of transcription, DNA-templated<br>negative regulation of Wnt signaling pathway involved in dorsal/ventral axis specification<br>neural crest cell fate commitment<br>osteoblast differentiation<br>planar cell polarity pathway involved in neural tube closure<br>positive regulation of canonical Wnt signaling pathway<br>positive regulation of cell growth<br>positive regulation of epithelial cell proliferation<br>positive regulation of extrinsic apoptotic signaling pathway via death domain receptors<br>positive regulation of fat cell differentiation<br>positive regulation of fibroblast apoptotic process<br>positive regulation of non-canonical Wnt signaling pathway<br>positive regulation of smoothened signaling pathway<br>positive regulation of transcription, DNA-templated<br>prostate epithelial cord arborization involved in prostate glandular acinus morphogenesis<br>proteolysis<br>regulation of branching involved in prostate gland morphogenesis<br>regulation of cell cycle process<br>regulation of dopaminergic neuron differentiation<br>response to drug<br>somatic stem cell population maintenance<br>stromal-epithelial cell signaling involved in prostate gland development<br>ureteric bud development<br>Wnt signaling pathway involved in somitogenesis

CHAAL00000009359	CTNNB1	Catenin Beta 1	Qinghai Lake: Tibetan plateau	Autosomes	adherens junction assembly<br>anterior/posterior axis specification<br>bicellular tight junction assembly<br>bone resorption<br>branching involved in ureteric bud morphogenesis<br>canonical Wnt signaling pathway involved in negative regulation of apoptotic process<br>canonical Wnt signaling pathway involved in positive regulation of cardiac outflow tract cell proliferation<br>canonical Wnt signaling pathway involved in positive regulation of epithelial to mesenchymal transition<br>cell fate specification<br>cell maturation<br>cell morphogenesis involved in differentiation<br>cell-matrix adhesion<br>cellular response to growth factor stimulus<br>cellular response to indole-3-methanol<br>central nervous system vasculogenesis<br>chemical synaptic transmission<br>chromatin-mediated maintenance of transcription<br>cranial ganglion development<br>dopaminergic neuron differentiation<br>dorsal root ganglion development<br>dorsal/ventral axis specification<br>ectoderm development<br>embryonic axis specification<br>embryonic brain development<br>embryonic cranial skeleton morphogenesis<br>embryonic digit morphogenesis<br>embryonic foregut morphogenesis<br>embryonic forelimb morphogenesis<br>embryonic heart tube development<br>embryonic hindlimb morphogenesis<br>embryonic skeletal limb joint morphogenesis<br>endodermal cell fate commitment<br>endothelial tube morphogenesis<br>epithelial cell differentiation involved in prostate gland development<br>epithelial tube branching involved in lung morphogenesis<br>fungiform papilla formation<br>gastrulation with mouth forming second<br>genitalia morphogenesis<br>glial cell fate determination

					hair follicle morphogenesis<br>hair follicle placode formation<br>hindbrain development<br>in utero embryonic development<br>layer formation in cerebral cortex<br>lens morphogenesis in camera-type eye<br>lung cell differentiation<br>lung induction<br>lung-associated mesenchyme development<br>male genitalia development<br>mesenchymal cell proliferation involved in lung development<br>metanephros morphogenesis<br>midbrain development<br>negative regulation of apoptotic signaling pathway<br>negative regulation of cell proliferation<br>negative regulation of chondrocyte differentiation<br>negative regulation of mesenchymal to epithelial transition involved in metanephros morphogenesis<br>negative regulation of mitotic cell cycle, embryonic<br>negative regulation of neuron death<br>negative regulation of oligodendrocyte differentiation<br>negative regulation of osteoclast differentiation<br>negative regulation of protein sumoylation<br>negative regulation of transcription from RNA polymerase II promoter<br>nephron tubule formation<br>neural plate development<br>neuron migration<br>odontogenesis of dentin-containing tooth<br>oocyte development<br>osteoclast differentiation<br>oviduct development<br>pancreas development<br>patterning of blood vessels<br>positive regulation of chromatin-mediated maintenance of transcription<br>positive regulation of determination of dorsal identity<br>positive regulation of DNA-templated transcription, initiation<br>positive regulation of endothelial cell differentiation<br>positive regulation of epithelial cell proliferation involved in prostate gland development<br>positive regulation of fibroblast growth factor production

					positive regulation of fibroblast growth factor receptor signaling pathway<br>positive regulation of heparan sulfate proteoglycan biosynthetic process<br>positive regulation of I-kappaB kinase/NF-kappaB signaling<br>positive regulation of MAPK cascade<br>positive regulation of mesenchymal cell proliferation<br>positive regulation of neuroblast proliferation<br>positive regulation of neuron apoptotic process<br>positive regulation of osteoblast differentiation<br>positive regulation of sequence-specific DNA binding transcription factor activity<br>positive regulation of skeletal muscle tissue development<br>positive regulation of telomerase activity<br>positive regulation of telomere maintenance via telomerase<br>positive regulation of transcription from RNA polymerase II promoter<br>protein localization to cell surface<br>proximal/distal pattern formation<br>regulation of calcium ion import<br>regulation of centriole-centriole cohesion<br>regulation of centromeric sister chromatid cohesion<br>regulation of core promoter binding<br>regulation of euchromatin binding<br>regulation of myelination<br>regulation of nephron tubule epithelial cell differentiation<br>regulation of protein localization to cell surface<br>regulation of secondary heart field cardioblast proliferation<br>regulation of smooth muscle cell proliferation<br>regulation of T cell proliferation<br>renal inner medulla development<br>renal outer medulla development<br>renal vesicle formation<br>response to drug<br>response to estradiol<br>smooth muscle cell differentiation<br>sympathetic ganglion development<br>synapse organization<br>synaptic vesicle transport<br>T cell differentiation in thymus<br>thymus development<br>trachea formation<br>transcription, DNA-templated

CHAAL00000006167	ATP1B1	ATPase Na+/K+ Transporting Subunit Beta 1	Qinghai Lake: Tibetan plateau	Autosomes	ATP hydrolysis coupled transmembrane transport<br>ATP metabolic process<br>cardiac muscle contraction<br>cellular calcium ion homeostasis<br>cellular potassium ion homeostasis<br>cellular sodium ion homeostasis<br>establishment or maintenance of transmembrane electrochemical gradient<br>membrane repolarization<br>mitophagy in response to mitochondrial depolarization<br>positive regulation of ATPase activity<br>positive regulation of calcium:sodium antiporter activity<br>positive regulation of defense response to virus by host<br>positive regulation of potassium ion import<br>positive regulation of potassium ion transmembrane transporter activity<br>positive regulation of sodium ion export from cell<br>potassium ion import<br>protein localization to plasma membrane<br>protein stabilization<br>regulation of cardiac muscle contraction by calcium ion signaling<br>regulation of gene expression<br>relaxation of cardiac muscle<br>sodium ion export from cell<br>xenophagy
CHAAL00000006574	IGFBP2	Insulin Like Growth Factor Binding Protein 2	Bohai Bay: Coastal	Autosomes	positive regulation of activated T cell proliferation<br>regulation of cell growth<br>regulation of insulin-like growth factor receptor signaling pathway
